# The relationship between gene traits and transcription in soil microbial communities varies by environmental stimulus

**DOI:** 10.7717/peerj.20641

**Published:** 2026-02-05

**Authors:** Peter F. Chuckran, Steven J. Blazewicz, Javier A. Ceja-Navarro, Jennifer Pett-Ridge, Egbert Schwartz, Paul Dijkstra

**Affiliations:** 1 Department of Environmental Science, Policy, and Management, University of California, Berkeley, Berkeley, United States; 2 Center for Ecosystem Science and Society (ECOSS) and Department of Biological Sciences, Northern Arizona University, Flagstaff, AZ, United States; 3 Physical and Life Sciences Directorate, Lawrence Livermore National Laboratory, Livermore, California, United States; 4 Lawrence Berkeley National Laboratory, Biological Systems and Engineering Division, Berkeley, California, USA; 5 Life & Environmental Sciences Department, University of California Merced, Merced, California, United States; 6 Innovative Genomics Institute, University of California Berkeley, Berkeley, California, United States

**Keywords:** Soil, Metatranscriptomics, Codon bias, Gene traits, Metagenomics

## Abstract

Codon and nucleotide frequencies are known to relate to the rate of gene transcription, yet how these traits shape transcriptional profiles of soil microbial communities remains unclear. Here we test the prediction that functional genes with high codon optimization and energetically lower cost nucleotides (*i.e*., nucleotides requiring less adenosine triphosphate (ATP) for synthesis) have higher transcriptional expression in a soil microbial community. In laboratory incubations, we subjected an agricultural soil to two separate short-term environmental changes: labile carbon (glucose) addition or a sudden 30-min increase in temperature from 20 °C to 60 °C. Using the total genomic codon frequencies to predict preferred codon usage for each taxon, we then estimated codon optimization for each transcript. On the community level, we found a higher average level of codon optimization after the addition of glucose. Synonymous nucleotide composition in the transcript pool also shifted towards energetically cheaper nucleotides, favoring uracil (U) over adenine (A) and cytosine (C) over guanine (G). Similarly, we found that encoded amino acid usage shifted towards energetically cheaper amino acids in response to labile carbon. In contrast, in communities responding to heat shock, there were no significant differences in the averaged gene traits of expressed transcripts. We used metagenome-assembled-genomes to further examine the ability of gene traits to predict transcriptional responses within and between taxa. We found that traits of individual genes could not reliably predict the level of transcription of a gene within or between taxa—highlighting the limits of this approach. However, we did find that when traits were averaged across several related genes, codon optimization was able to predict levels of transcription in metabolic pathways associated with growth and nutrient uptake in response to glucose. Similar relationships were not observed in response to heat, or for functions associated with stress—such as genes associated with sporulation or heat shock. These results demonstrate that gene traits, such as codon usage, nucleotide selection, and amino acid selection, relate to the transcriptional expression of genes in soil microbial communities and suggests that these relationships may be dependent on both gene function and the specific type of environmental stimuli.

## Introduction

Soil microbes are often subject to short term changes in their environment which exert a strong influence on community structure and function (Schimel, Balser & Wallenstein, 2007; Griffiths & Philippot, 2013). Elucidating the factors that dictate the response of soil microbes to changes in their environment is therefore crucial for assessing both their immediate activity and long-term ecological roles (Schimel, Balser & Wallenstein, 2007; Shade et al., 2012). Gene traits such as codon bias, nucleotide frequency, and amino acid frequency are known to influence microbial gene expression in culture (Chen et al., 2016; Zhou et al., 2016) and may represent valuable, yet underexplored, metrics for predicting responses of soil microbial communities to changes in the environment. Although studies have examined how short-term environmental changes influence gene expression in soil microbial communities (Peng, Wegner & Liesack, 2017; Albright et al., 2018; Chuckran et al., 2021; León-Sobrino et al., 2024), the influence of gene-specific traits on the transcription of genes in soils remains unclear.

Codon optimization, defined as the high alignment of gene codon frequencies to the anticodons of the tRNA pool, is generally associated with greater gene expression as a result of higher rates of both translation and transcription (Plotkin & Kudla, 2011). During translation, the level of codon optimization of a transcript directly impacts protein production by altering the rate of elongation, protein folding, initiation, and termination (Zhou et al., 2013; Yu et al., 2015; Liu, Yang & Zhao, 2021). During transcription, codon usage and nucleotide selection directly influences transcript abundance by altering mRNA stability (Dressaire et al., 2013; Presnyak et al., 2015), the differential energetic cost of synonymous nucleotide synthesis (Chen et al., 2016), and the energetic cost of dsDNA unwinding (Villada, Duran & Lee, 2020). The relationship between transcription rate and codon usage is not entirely causal, rather, it is also the product of selection for optimized codons on highly upregulated genes (Seward & Kelly, 2016).

Due to the relationship between codon usage and gene expression, codon frequencies represent a valuable trait for assessing life strategies in microbial communities. For example, codon usage has been used to predict growth rates of individual bacterial taxa in microbial communities (Weissman, Hou & Fuhrman, 2021). It has also been shown that greater codon optimization is associated with higher growth rates and copiotrophic life strategies in soil (Chen et al., 2021). Similarly, high levels of codon bias (often highly correlated with codon optimization; Bahiri-Elitzur & Tuller, 2021) in ribosomal protein genes was shown to be associated with rapid transcriptional responses to rewetting in soil microbial communities (Chuckran et al., 2025). However, the relationship between codon bias and transcription for non-ribosomal genes in soil communities is relatively unexplored.

Gene expression is also related to the energy and nutrient cost of nucleotide and amino acid synthesis. For example, thymine (T) requires less energy to synthesize then adenine (A), cytosine (C) requires less energy than guanine (G), and G+C base pairs are more costly to produce than A+T (Chen et al., 2016). For this reason, highly upregulated genes tend to exhibit lower AT and GC skew on the sense strand (*i.e*., more T than A, more C than G; Chen et al., 2016). Despite the higher cost of synthesis, a high GC content has been associated with elevated levels of gene expression in bacteria. There are likely several reasons for this, one being that high GC codons tend to encode for energetically cheaper amino acids which often have a higher level of expression (Raiford et al., 2012). Additionally, high GC content correlates with high levels of codon bias, which is itself linked to enhanced gene expression efficiency (Plotkin & Kudla, 2011).

Other gene-level attributes, such as amino acid composition, have also been shown to be associated with transcriptional response. Nutritional constraints influence amino acid composition for corresponding transporters (Baudouin-Cornu et al., 2001), potentially affecting their rate of transcription. For example, under nitrogen limitation, transporters that require less nitrogen for synthesis are more likely to be transcribed (Read et al., 2017). Since there is a positive relationship between the C:N of amino acids and the C:N of the nucleotides of their associated codons (Sueoka, 1961; Bragg & Hyder, 2004; Shenhav & Zeevi, 2020), gene sequences that conserve limiting nutrients may be more highly upregulated in response to changes in nutrient availability. Although there is evidence for a relationship between nutrient conservation and nucleotide selection in soils (Chuckran et al., 2023), it is not yet known how these traits are related to the short-term transcriptional response of soil microbial communities.

In a previous study, we showed that the addition of labile carbon in the form of glucose can rapidly stimulate the transcription of nitrogen cycling genes (Chuckran et al., 2021). Since soil heterotrophic bacteria are generally carbon-limited (Demoling, Figueroa & Bååth, 2007; Hobbie & Hobbie, 2013), the addition of a labile carbon source rapidly stimulates microbial activity, leading to rapid immobilization of available nitrogen (Kamble & Bååth, 2014; Chuckran et al., 2021). Here we present an analysis of this experiment with a focus on codon frequency, nucleotide composition, and amino acid composition. We also analyzed metatranscriptomes from a heat-stress experiment, where soils were heated from 20 °C to 60 °C for 30 min. We hypothesized that, in response to both sudden environmental changes, highly and rapidly expressed genes would have higher codon optimization. We also hypothesized that nucleotide and amino acid composition in these transcripts would reflect both energy and nutrient conservation, with nucleotides and amino acids that are cheaper to produce being more prevalent in the rapidly responding transcript pool. Through this analysis, we aim to improve our understanding of how codon usage, nucleotide composition, and amino acid content can predict the potential responses of soil microbial communities to perturbations such as carbon addition or higher temperatures.

## Materials and Methods

### Soil collection

Soils were collected from the West Virginia Certified Organic Farm (Morgantown, West Virginia, USA, 39.647502°N, 79.93691°W; 243.8 to 475.2 m above sea level) in the fall of 2017. Soils were sampled from plots subject to a four-year conventionally tilled crop cycle of corn, soybean, wheat, and a mix of kale and cowpea, with manure additions every 2 years and a rye-vetch winter crop cover (Pena-Yewtukhiw et al., 2017; Walkup et al., 2020). The study area contains predominately silt-loam soils, where site-averaged pH was 6.54 and soil organic matter was 46.4 g kg^−1^ (Pena-Yewtukhiw et al., 2017; Walkup et al., 2020). Ten cores 0–10 cm in depth were collected from each plot and pooled. Soils were shipped on ice to Northern Arizona University (Flagstaff, Arizona, USA). An equal amount of soil from each plot was pooled and passed through a 2 mm sieve, with large roots and debris being manually removed.

### Temperature experiment

Pooled soils were distributed in 10 glass Mason jars (500 ml) at 30 g of soil each. Samples were preincubated at ~23 °C for 2 weeks. The lid of each jar was briefly opened after preincubation to refresh the headspace of the jar before treatment. Five of the samples were placed in an incubator at 20 °C and five samples were placed at 60 °C for 30 min. Soils were then destructively sampled and immediately frozen in liquid N_2_ to preserve the nucleic acids.

### Glucose addition experiment

Pooled soil was added to 65 glass Mason jars (500 ml) at 30 g of soil each and preincubated at ~23 °C for 2 weeks. After preincubation, 1.6 mL of 0.13 M glucose (0.7 mg glucose C g^−1^ dry soil) was added to 60 of the samples. Five samples were left untreated as controls (*t*_*0*_). Every 4 h for 48 h, the CO_2_ concentration in the headspace of each jar was measured and five chambers were destructively sampled for a total of 13 timepoints (including *t*_*0*_). During destructive sampling, a portion of the soil was frozen in liquid N for nucleotide extractions, and another portion was reserved to measure concentrations of NO_3^−^_, NH_4^+^_, and microbial biomass. A more extended description of the soil collection and glucose addition experiment can be found in Chuckran et al. (2021).

### Metagenomes and metatranscriptomes

RNA and DNA were extracted from four samples for each temperature (20 °C and 60 °C) and from four samples of four timepoints from the glucose incubation: 0 (*t*_*0*_), 8 (*t*_*8*_), 24 (*t*_*24*_), and 48 h (*t*_*48*_), using the RNeasy PowerSoil total RNA kit (Qiagen) and RNeasy PowerSoil DNA elution kit (Qiagen), respectively. DNase was removed using an RNase-free DNase set (Qiagen). A Qubit fluorometer (Invitrogen, Carlsbad, CA, USA) was used to measure nucleotide concentrations and NanoDrop ND-1000 spectrophotometer (Nanodrop Technologies, Wilmington, DE, USA) was used to assess sample purity. Samples were shipped to the Joint Genome Institute for sequencing on an Illumina NovaSeq platform (San Diego, CA, USA).

Sequence data was processed by the Joint Genome Institute (JGI). A detailed description of the sequencing and bioinformatics pipeline can be found in the associated data release (Chuckran et al., 2020) and accession numbers for the heat-shock experiment can be found in Table S1. Briefly, raw sequences were QC filtered using BBtools v.38 (Bushnell, 2014). Metatranscriptomes were assembled with MEGAHIT v.1.1.2 (Li et al., 2015) and metagenomes were assembled using SPAdes v3.13.0 (Bankevich et al., 2012). Coverage against assembled contigs was determined using BBMap v38 (Bushnell, 2014). Contigs were annotated with the IMG Annotation Pipeline v 4.16.5 (Huntemann et al., 2016; Chen et al., 2019). The following analyses use annotations from the IMG Annotation Pipeline, with functional annotations against the KEGG database (Kanehisa & Goto, 2000) and taxonomic annotations against the IMG database.

To assess the influence of nucleotide and codon frequency on transcription as they relate to function, several of our analyses focus on groups of KO numbers which were derived from KEGG pathways or gene descriptions. Functional groups included: carbon metabolism, nitrogen assimilation, ribosomal proteins, and sporulation. For genes associated with carbon metabolism and ribosomal proteins, we used genes in the KEGG pathways “ko01200” and “ko03010” respectively. For sporulation genes, we searched for genes which contained “spore” or “sporu” in the gene description and manually inspected the gene descriptions to assure they were associated with sporulation. For nitrogen assimilation genes, we used a curated list, including genes involved in: assimilatory nitrate reduction (K00360, K00366, K00367, K00372, K10534, K17877), glutamate dehydrogenase (GDH; K00260, K00261, K00262), glutamine synthetase and glutamate synthase (GS-GOGAT; K00264, K00265, K00266, K00284, K01915), nitrate assimilation (K02575, K15576, K15577, K15578, K15579), nitrogen fixation (K00531, K02586, K02588, K02591, K22896, K22897, K22898, K22899), ammonium transport (K03320), and nitrogen regulatory proteins (K04751, K04752, K07708, K07712, K00990, K19338, K03719, K00982, K03092).

### Metagenome assembled genomes

To further assess the power of genomic traits for predicting responses between taxa, we generated a reference set of metagenome-assembled-genomes (MAGs) and mapped metatranscriptomes to this references set of MAGs. Whereas the community-averaged traits derived from contigs allow for the examination of broad trends in the relationship between genomic traits of the transcript pool, comparing traits and expression on the MAG-level provide a more-accurate assessment of how well traits might predict expression for specific functions within or between taxa.

To bin contigs, we selected the metagenome with the largest assemblies from each treatment and mapped all other QC-filtered metagenomic short-reads to that set of contigs using bbmap (flags: minid = 0.97 ambiguous = random). We then used COMEBin (Wang et al., 2024) using the default parameters to bin contigs. Medium and high quality MAGs (>50% complete, < 10% contamination) from each metagenome were then dereplicated using dRep (Olm et al., 2017) with the flag-comp 50. QC-filtered metatranscriptome short-reads were mapped against the reference set of contigs using bbmap (flags: minid = 0.99 ambiguous = random). From the gene calls and annotations generated by JGI, the transcript counts for each gene was calculated using featureCounts (Liao, Smyth & Shi, 2014) and then normalized using DESeq2 (Love, Huber & Anders, 2014). The taxonomic identity of each MAG was determined against the GTDB database using GTDB-tk (Chaumeil et al., 2022) with the command “gtdbtk classify_wf –skip_ani_screen”.

### Calculating gene traits

We used four indices to calculate codon optimization: the Codon Adaptation Index (CAI; (Sharp & Li, 1987)), the Frequency of Optimized Codons (FOP; Ikemura, 1981), the Measurement Independent of Length and Composition (MILC; Supek & Vlahoviček, 2005), and the effective number of codons (ENC’, Novembre, 2002). Each of these indices requires a reference set of frequencies from which to calculate codon bias. Typically this would be generated using the anticodons of the tRNA pool (Bahiri-Elitzur & Tuller, 2021). However, we found that the tRNA annotations from both our metagenomes and metatranscriptomes did not cover a broad range of taxa, severely limiting our estimates of codon optimization. Instead, we used the genomic background codon frequencies of each taxon derived from the metagenomes. Although codon frequencies of the genomic background do not represent the tRNA pool, they should represent the preferred synonymous codon usage in the tRNA pool, and have been successfully used for calculating codon optimization in tools predicting growth rates in metagenomes (Weissman, Hou & Fuhrman, 2021). BEDTools (Quinlan & Hall, 2010) was used to isolate gene sequences in metagenomic contigs from general feature format files generated by the IMG analysis pipeline. Using the taxonomic annotations and read coverage, we determined the relative frequency of each codon for each taxon in our metagenomes. We then removed species with a total depth-adjusted codon count below 750,000, leaving a total of 720 species. We also summarized codon frequencies at the phylum level, recovering codon frequencies for 46 phyla. Taxon-specific codon frequencies were then used to calculate codon indices for each transcript in the metatranscriptomes at both the phylum and species level of annotated contigs, as well as for individual MAGs.

The Codon Adaptation Index (CAI) was calculated by first determining weights for each amino acid from the reference dataset (*i.e*., the taxon-level codon frequencies from the metagenomes):

(1) $${w_c} = {{{f_c}} \over {max({f_{synonymous\; codons)}}}},$$

where the weight of each codon, *w*_*c*_, is determined as the frequency at which it occurs in the genome, *f*_*c*_, divided by the maximum codon frequency of a synonymous codon for the encoded amino acid. For example, glutamic acid is encoded by GAA and GAG. If the codon frequencies for a genome were 0.2 for GAA and 0.8 for GAG, the weights would be 0.25 and 1. These reference weights were applied to each codon in a transcript. Building on the previous example, a transcript with the sequence “GAA GAA GAG” would yield a list of weights [0.25, 0.25, 1]. The geometric mean of these weights represents the CAI for that transcript, as shown in Eq. (2):

(2) $$CAI = \left( \prod \limits_{c = 1}^L {w_c}\right ){\; {{1 \over L}}},$$

where L is the length of the transcript, calculated as the number of codons. In the above example, the list of weights of [0.25, 0.25, 1], would produce a CAI value of 0.40. CAI values close to 1 indicate a high level of codon optimization.

Frequency of Optimized Codons (FOP) was calculated for each transcript as:

(3) $$FOP = {{Number\; of\; optimized\; codons} \over {Total\; number\; of\; codons}},$$

where the optimized codons were determined from the reference codon frequencies from the DNA for the corresponding taxon. Like CAI, values closer to 1 indicate a higher level of codon optimization. FOP and CAI values are generally well correlated (Bahiri-Elitzur & Tuller, 2021).

Measure Independent of Length and Composition (MILC; Supek & Vlahoviček, 2005) values were calculated by first calculating codon bias for each amino acid, *M*_*a*_, as in Eq. (4):

(4) $${M_a} = \sum {O_c}\; ln\displaystyle{{{f_c}} \over {{g_c}}}$$

where *O*_*c*_ is the count of codon *c*, *f*_*c*_ is the observed frequency of codon *c* for amino acid *a* in the transcript, and *g*_*c*_ is the expected frequency of codon *c* of amino acid *a*. MILC is then calculated as in Eq. (5):

(5) $$MILC = {{ \sum \nolimits_a {M_a}} \over L} - C$$

where *L* is the gene length in codons and *C* is a correction for overall bias in short sequences as calculated in Eq. (6):

(6) $$C = {{ \sum \nolimits_a \left( {{r_a} - 1} \right)} \over L} - 0.5$$

where *r*_*a*_ is the total number of synonymous codons for amino acid *a*. MILC values closer to 0 indicate a higher level of optimization.

FOP, CAI, and MILC values were calculated for each transcript using either phylum-level (for contig-based analyses) or MAG-level reference codon frequencies of the background DNA. Genes with fewer than 80 codons were discarded to minimize bias due to low frequencies. Codon frequency and bias calculations were conducted using custom-made Python scripts which are available at https://doi.org/10.6084/m9.figshare.30446078. ENC’ values were calculated only for MAGs using the function ENCprime in gRodon (Weissman, Hou & Fuhrman, 2021).

Using the amino acid sequence provided by the IMG annotation pipeline, we summed the number of each amino acid for all genes. Using the chemical formula of the amino acids, we then calculated the total stoichiometric ratio for each transcript. Similar to methods described in Chen et al. (2016), we used the total number of phosphate bonds used for synthesis, derived from Akashi & Gojobori (2002) (Table S2), to calculate the metabolic cost per amino acid, which we then used to calculate the mean cost per amino acid for each transcript.

Nucleotide frequencies, such as GC content, GC-skew, and AT-skew, were calculated for each gene. GC-skew was calculated as:

${\rm GC\; skew} = {\rm \; }\displaystyle{{G - C} \over {G + C}}.$

And AT-skew as:

${\rm AT\; skew} = { \; }{{A - T} \over {A + T}}.$

Nucleotide frequencies at fourfold degenerative sites (*i.e*., where any substitution at the site would encode for the same amino acid), were used as a metric of synonymous nucleotide frequencies. Non-synonymous frequencies were calculated from sites where any substitution would change the encoded amino acid.

Indices, nucleotide content, and amino acid content were adjusted for read depth and summarized by either KEGG Orthology (KO) number (Kanehisa & Goto, 2000) and taxa; KO number and phylum; or KO number alone. Data was summarized using the python library pandas (The Pandas Development Team, 2025).

### Statistical analyses

For community-level analyses, differences in transcription between treatments, calculated as log_2_-fold change (LFC), were determined using DESeq2 (Love, Huber & Anders, 2014) using the total number of counts for each function (*i.e*., KO number). In MAGs, differential expression was determined using DESeq2 for individual genes. In both the analysis of community-averaged and MAG-level transcription, a Wald-test was used to determine differences in gene expression between treatments and a false discover rate (FDR) of < 0.1 indicated significance differences in the expression of a gene between two timepoints or treatments. Differences in codon indices, GC, and amino acid content between treatments were determined using analysis of variance (ANOVA). The relationships between LFC and genomic traits were determined for each treatment using multiple linear regression. Nondimensional multidimensional scaling (NMDS) was performed using the vegan package (v 2.7.1, Oksanen et al., 2025). All statistical analyses were conducted in R version 4.1.0 (Team, 2018) and visualized with the ggplot2 package (Wickham, 2016).

## Results

**Figure 1:**
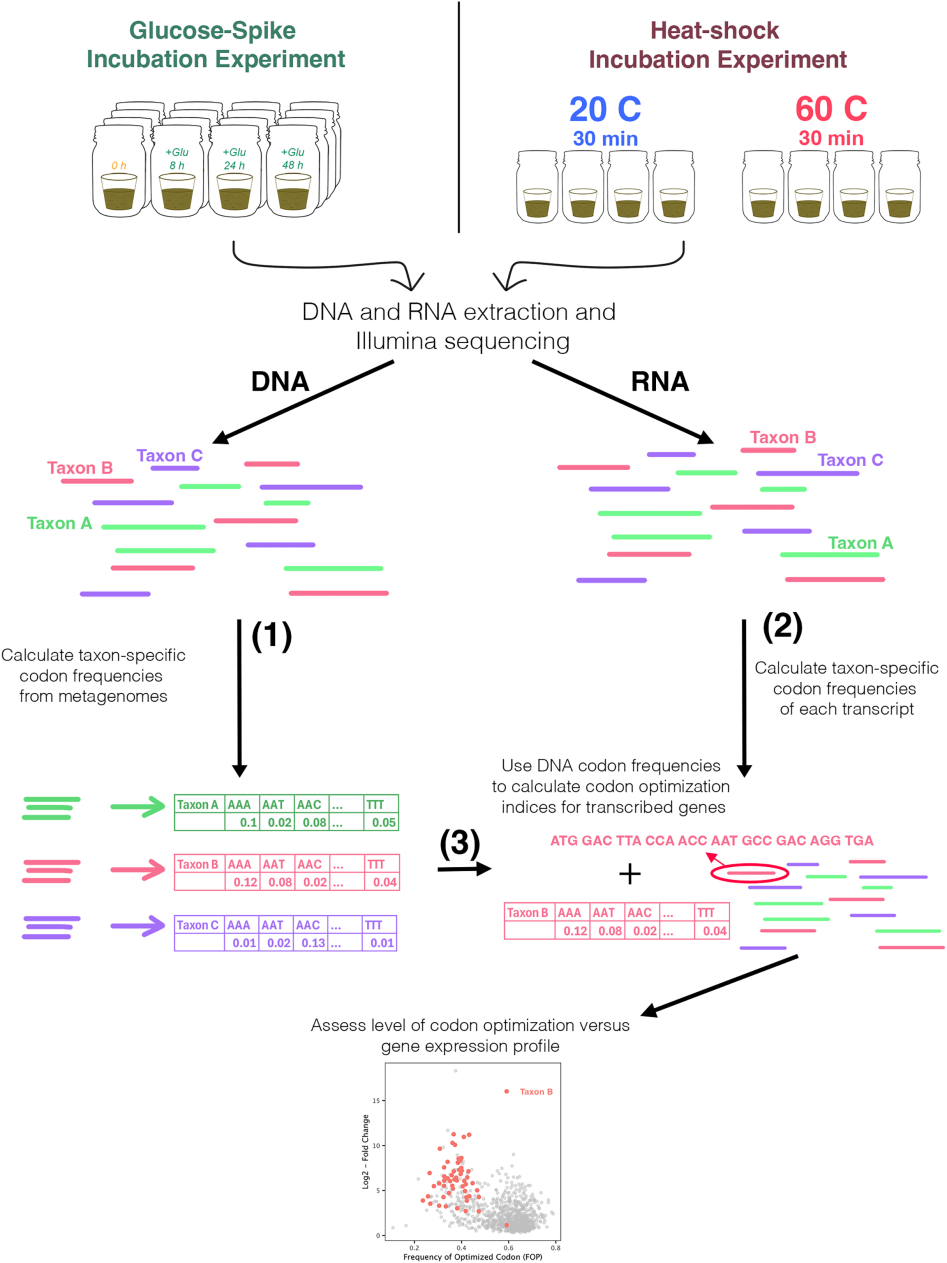
Overview of glucose-addition and heat-shock experiments and analysis. Featured analyses used metatranscriptomes from two different experiments: an incubation observing the community response to glucose additions at four timepoints (0, 8, 24, and 48 h); and in response to a sudden increase in temperature—30 min at 60 °C *vs*. a 20 °C control. Codon frequencies were calculated for all genes in each taxon and were then used to calculate codon optimization metrics for each functional genes of that taxon. These values were then used to assess the relationship between codon optimization and gene expression.

We conducted labile carbon addition and heat shock incubations to investigate microbial transcriptional responses under conditions of carbon enrichment and thermal stress (Fig. 1). Addition of labile carbon caused a rapid immobilization of both soil carbon and nitrogen, as measured by CO_2_, NO_3^−^_ and NH_4^+^_ (described in Chuckran et al. (2021)). Nitrate decreased from 2.01 μg NO_3^−^_-N gram^−1^ soil to 0.25 μg NO_3^−^_-N gram^−1^ soil over the 48 h incubation, ammonium decreased from 0.28 to 0.19 μg NH_4^+^_-N gram^−1^ soil, and soluble C decreased from 44.1 to 4.4 μmol extractable organic C g^−1^ soil.

### Community-averaged results

In response to the addition of glucose, we found an upregulation of 2,549 and downregulation of 1,273 KO functions (FDR < 0.1). An increase in temperature from 20 °C to 60 °C for 30 min resulted in the upregulation of 79 and the downregulation of 15 KO functions (FDR < 0.1).

**Figure 2:**
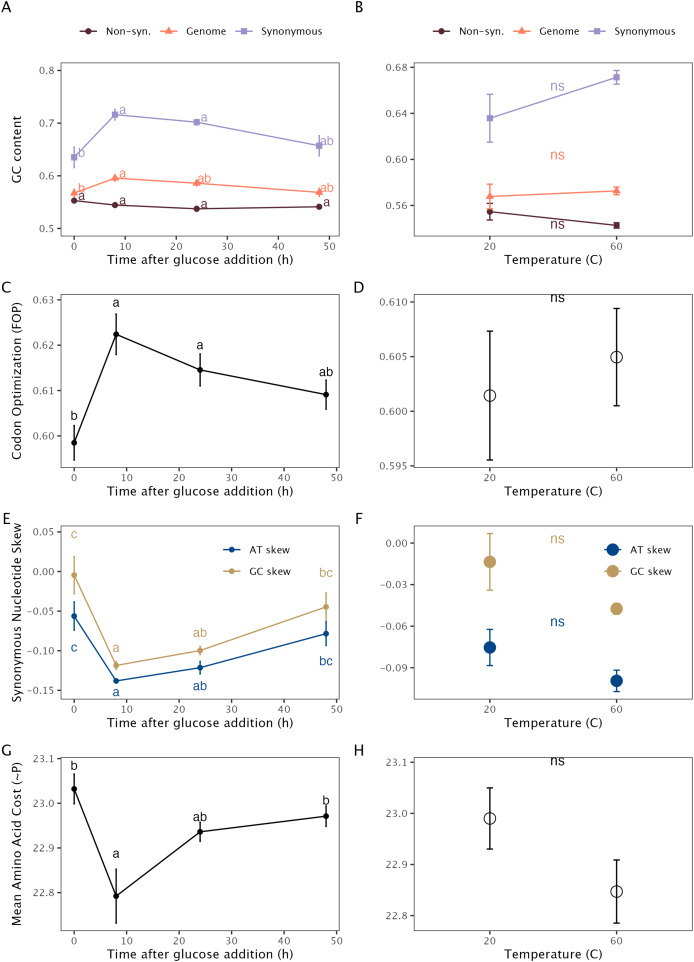
Averaged gene traits of metatranscriptomes after addition of glucose and heat-shock. Average gene traits of metatranscriptomes (*n* = 4) in glucose-spike incubations (left) and a heat-shock experiment (right): (A & B) GC content for all genes (orange), synonymous substitution sites (purple), and non-synonymous substitution sites (burgundy); (C & D) codon optimization as calculated by MILC, and displayed as (1-MILC), such that higher values represent a higher frequency of optimized codons; (E & F) nucleotide skew (calculated for synonymous sites; and (G & H) the mean cost of encoded amino acids, as measured by the total number of phosphate bonds required for synthesis.

The GC content of the total transcript pool increased in response to glucose addition (Fig. 2A). This trend was driven by shifts at synonymous substitutions sites, where we observed a large increase in GC content after the glucose addition, whereas there was no significant shift in the GC content at non-synonymous substitution sites (Fig. 2A). Metatranscriptome-level indices of codon optimization increased with the addition of glucose across all indices at 8 h (Tukey’s HSD; *p* < 0.05; Fig. 2C). We also found a significant decrease in the GC-skew ${\left({G - C} \over {G + C}\right)}$ and AT-skew ${\left({A - T} \over {A + T}\right)}$ at synonymous substitution sites (Fig. 2E)—although not at nonsynonymous sites (Fig. S1). We did not find any significant shifts in the metatranscriptome-level nucleotide frequencies or codon optimization indices in the heat-shock experiment (Figs. 2B, 2D, & 2F). Metatranscriptomic analysis showed that the average cost of amino acid synthesis decreased after glucose addition but did not significantly change with increased temperature (Figs. 2G & 2H).

**Figure 3:**
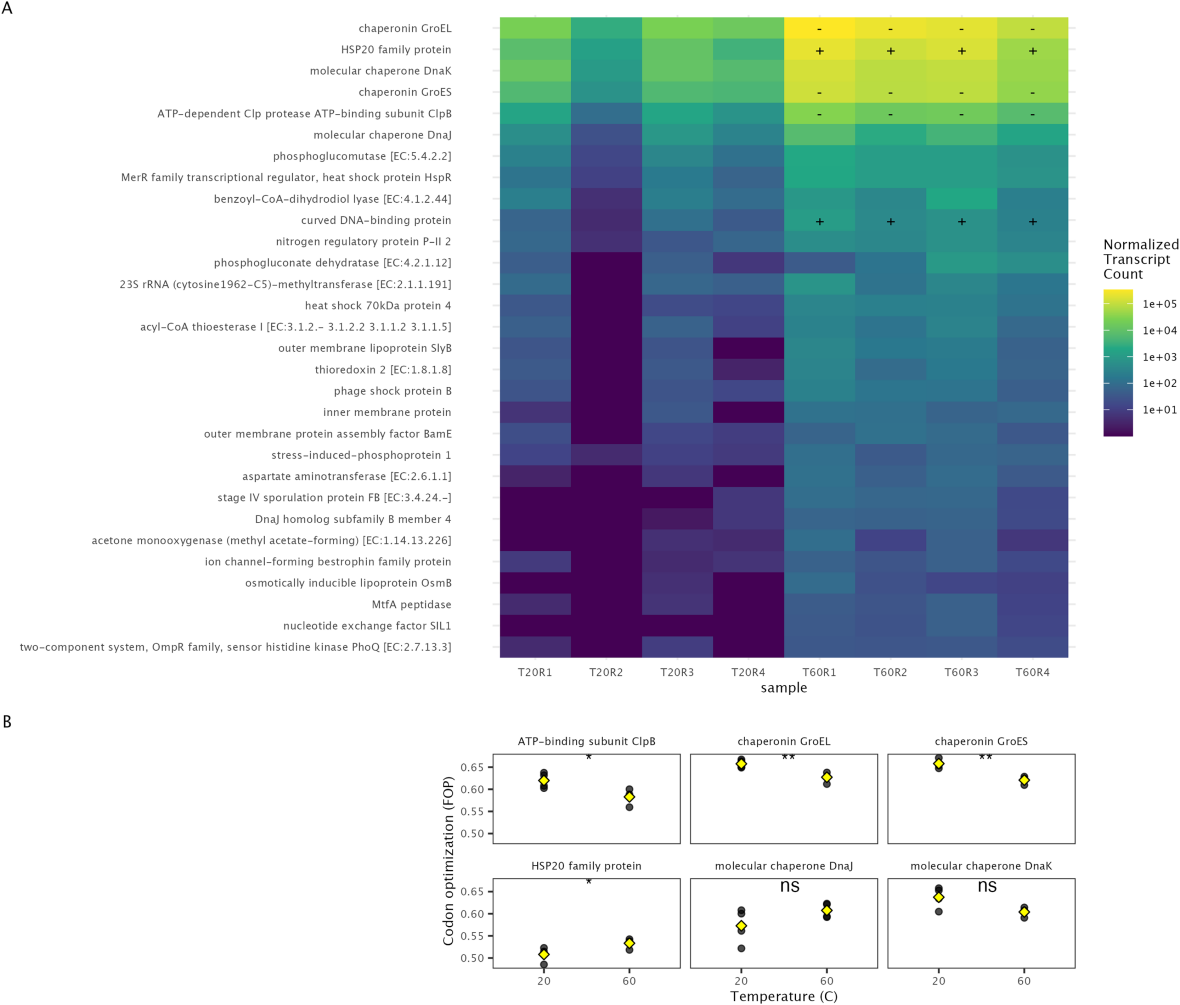
Codon optimization of highly expressed genes in response to heat shock in soil microbial communities. The gene counts (color) of the top 30 most abundant upregulated functions after a temperature increase in the heat-shock experiment, (A). Boxes marked with a (−) indicate the average codon optimization for that function significantly decreased from 20 °C to 60 °C, a (+) marks an increase. (B) The average codon optimization (FOP) within a metatranscriptome of top 6 most abundant upregulated genes, with black points representing average of each metatranscriptome, and yellow diamond representing the mean between samples.

A majority of highly upregulated genes in the heat-shock experiment encoded for chaperones and heat-shock proteins (Fig. 3A). We found that the average codon optimization for genes with these KO numbers either did not change or significantly decreased in response to heat-shock (Figs. 3A & 3B).

### MAG-level results

We binned 53 medium to high-quality MAGs (>50% complete) to test the ability of gene traits to predict transcriptional expression of genes within and between genomes. Within a genome, we did not find strong evidence that upregulated genes had significantly different features than down or non-differentially expressed genes. Levels of optimization were consistently higher in upregulated genes (Fig. S2); however, the significance of this relationship was inconsistent between codon usage metrics. Traits such as synonymous nucleotide usage were generally not significantly different with transcriptional expression (ANOVA: GC skew, *p* = 0.316; GC content *p* = 0.991), with the one exception being AT-skew at *t*_*8*_, wherein upregulated genes had lower AT skew (ANOVA: *p* = 0.0179). Mean amino acid cost and C:N did not significantly vary in up *vs*. down regulated genes (ANOVA: *p* = 0.192 and *p* = 0.425, respectively).

Using a set of highly upregulated genes related to C and N cycling (*glnA*-K01915, *gltD*-K00266, amt-K03320, *glnD*-K00990), we tested the relationship between codon optimization and transcriptional expression on a per-function basis between MAGs and found no consistent relationship (Fig. S3).

**Figure 4:**
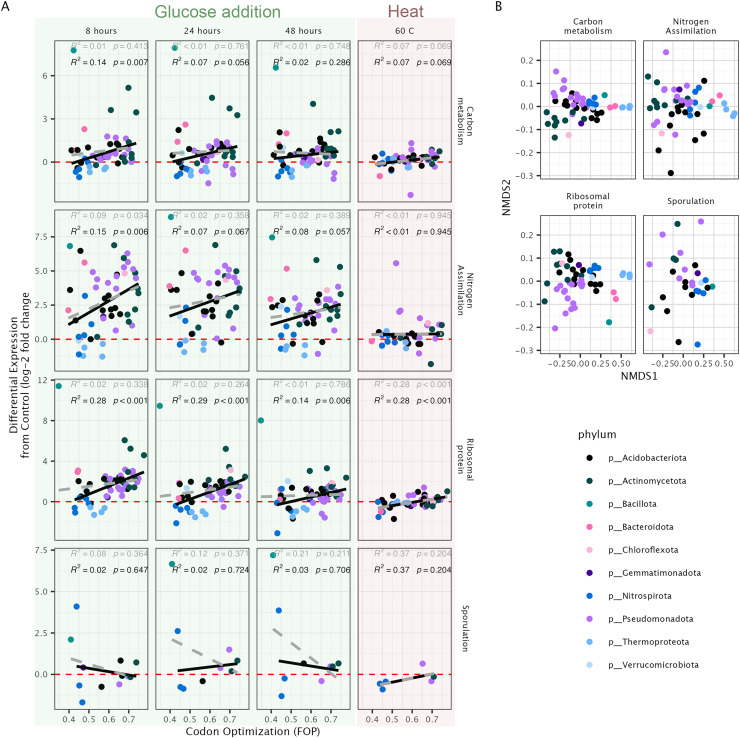
Transcriptional expression of metagenome assembled genomes in response to the addition of glucose and heat shock. Expression and codon usage in metagenome assembled genomes (MAG) in response to glucose addition or heat-shock. (A) The relationship between codon optimization, represented here as the frequency of optimized codon (FOP) *vs*. differential transcriptional expression from control samples (log_2_-fold change). Separated by pathway (rows) and treatment (columns). (B) Nonmetric multidimensional scaling (NMDS) of codon frequencies of each MAG for select pathways.

We found relationships between codon usage and average transcriptional expression in response to glucose when gene traits were summarized across all genes in a pathway. Specifically, 8 h after the addition of glucose we found a positive relationship between optimization and transcriptional expression in genes associated with carbon metabolism, nitrogen uptake, and ribosomal proteins (Fig. 4). This relationship was significant for ribosomal protein genes at *t*_*24*_ and *t*_*48*_ as well as during the heat-shock experiment—however, the slope of this relationship was larger in response to glucose than with heat shock. The observed relationships held for several codon bias metrics (Fig. S4). The significance of these relationships was often strongly impacted by a lone representative of the phylum Bacillota—which we found to have extremely high levels of differential transcriptional expression compared to other taxa (Fig. 4).

## Discussion

Our study examined the transcriptional responses of soil microbial communities to labile carbon addition and heat shock, revealing distinct strategies for coping with environmental perturbations. Glucose addition triggered widespread gene upregulation, accompanied by shifts in nucleotide composition, codon optimization, and amino acid cost—demonstrating that these traits are associated with rapid growth and resource efficiency. In contrast, heat shock elicited a narrower response, primarily activating stress-related genes without significant shifts in metatranscriptome-level nucleotide or codon usage patterns. These contrasting responses underscore the context-dependent nature of microbial transcriptional strategies.

After the addition of glucose, we found significant shifts in transcript nucleotide composition. At synonymous substitution sites—where the nucleotide used does not change the encoded amino acid—we found lower GC-skew ${\left({G - C} \over {G + C}\right)}$ and AT-skew ${\left({A - T} \over {A + T}\right)}$. Cytosine requires less energy to produce than guanine, and uracil/thymine require less energy than adenine, such that lower GC and AT skew at synonymous sites have been shown to be associated with higher levels of transcription (Chen et al., 2016). Similarly, in previous work observing the transcriptional response of soil metagenome-assembled genomes (MAGs) during rewetting, we found low levels of GC and AT-skew in fast-responding taxa (Chuckran et al., 2025). Together, these results demonstrate that the effect of nucleotide skew on transcription is detectable in soil microbial communities and may represent an important trait for rapid transcriptional response in soil microbes. Similarly, the energetic cost of amino acids encoded in transcripts (measured by the total number of phosphate bonds needed for synthesis) decreased after the addition of glucose, indicating that the use of both cheaper nucleotides and amino acids is associated with rapid transcriptional response.

Despite the higher energetic cost of the GC base pair *vs*. the AT base pair, we found that metatranscriptome GC-content increased after the addition of glucose. This effect was most pronounced at synonymous substitution sites, but non-existent at non-synonymous sites—suggesting that the increased codon optimization which occurs with greater GC content (Plotkin & Kudla, 2011) may be driving this relationship. In other words, higher levels of codon optimization are worth the increased energetic cost of the GC base pair and, accordingly, we found that the addition of glucose increased the average level of codon optimization in the transcript pool. This is similar to our previous findings in soil growth responses to rewetting, where we found that codon optimization and GC content were correlated such that the majority of growing bacterial taxa had a high GC content; however, those taxa which could maintain high optimization with lower GC content demonstrated the highest growth rates (Chuckran et al., 2025).

In our heat-shock experiment, gene traits averaged at the metatranscriptome level demonstrated similar but non-significant trends to the glucose-spike experiment (*e.g*., higher codon optimization, lower cost nucleotides and amino acids); however, none of the changes observed in the heat shock experiment were significant at *p* < 0.05. There has been little metatranscriptomic analysis of heat-shock in soil microbial communities, making it difficult to determine if our sampling (30 min at 60 °C) represented the optimal conditions to capture the heat-shock transcriptional response; although studies of bacteria in culture indicate that 30 min should fall in a range which captures the transcriptional response to heat-shock (Kim et al., 2020). It is also logical that heat-shock, a disturbance which would inhibit activity and growth, would not elicit the same response in either magnitude or direction as the addition of glucose. For example, 2,549 upregulated functions were upregulated on the community-level with the addition of glucose *vs*. 79 for heat shock. It is possible that relatively smaller transcriptional response to heat shock would therefore not result in the same metatranscriptome-level shift in nucleotide composition.

By examining gene-specific traits and transcription withing MAGs, we were able to assess the predictive power of gene traits both within and between genomes. Overall, we found that traits poorly predicted transcription within a genome. Although we did find that some metrics of codon optimization showed increased optimization in upregulated genes, this signal was not consistent among indices. This demonstrates a limitation in the use of nucleotide and codon frequencies for predicting activity within a genome. Trait relationships, although significant on the community-scale, might not be detectable on the scale of individual genes within a taxon.

The ability for traits to predict transcription between individual species was mixed. Traits could not predict transcription between taxa on the level of gene function; however, we did find several relationships when we averaged traits across all the genes in a metabolic pathway. Notably, we observed a relationship between codon optimization and transcriptional expression in genes related to carbon metabolism and nitrogen uptake, as well as for ribosomal protein genes. This result suggests that codon usage could be used to predict the activity of functional pathways in microbial communities, and more work should be done to link codon usage to short-term process rates in soils.

We observed notable exceptions in the relationship between codon optimization and transcriptional expression in specific taxa, environmental stimuli, and gene function. Taxonomically, we found that the lone representative of the phylum Bacillota was a consistent outlier in its level of transcription in response to glucose—where we observed low levels of optimization associated with very high levels of expression. One driver of this relationship perhaps lies in a key evolutionary difference in the replication machinery for this taxon. Many members of Bacillota have a unique alpha subunit of the DNA polymerase III encoded by *polC* as opposed to *dnaE* (Zhao et al., 2007)—which we also found in the KEGG annotations of this MAG (KO#: K03763). This subunit contains its own DNA repair mechanisms, which can both influence GC content (Zhao et al., 2007) and create unique codon usage signatures (Masłowska-Górnicz et al., 2022). Accordingly, in an assessment of codon usage patterns, we found that this Bacillota representative had unique codon usage frequencies in its ribosomal protein genes compared to other bacteria (Fig. 4B) and tended to be on the edge of the distribution of all other bacteria in codon frequencies for genes associated with carbon metabolism, nitrogen uptake, and sporulation (Fig. 4B). Further, *polC* also coevolved with its own degradosome, which may influence RNA turnover (Engelen et al., 2012). Combined, these results show key evolutionary differences which might influence the nature of the relationship between gene traits and expression—which represents an important consideration moving forward when attempting to connect traits to process rates.

We also found that genes not associated with nutrient uptake or growth, such as for sporulation, did not have the same relationship between codon optimization and transcription. Notably, there were fewer representative MAGs containing sporulation genes, limiting the predictive power of these traits for sporulation. Another possible influence on this relationship is that cells can adjust their tRNA pool in response to stress. Conditions, such as nutrient limitation, may increase the abundance of rare tRNA anti-codons, optimizing stress-response genes for translation under these altered conditions (Advani & Ivanov, 2019). To measure codon optimization in our study, we used the codon frequencies of the entire genome as a reference for the preferred codons for each amino acid—assuming that the codon abundance of a genome would generally align with the tRNA pool. Under normal growth conditions, this is a reasonable assumption (as indicated by the high levels of optimization in ribosomal protein genes). However, as a result of relying on DNA codon usage for reference frequencies, sporulation genes adapted to a stress-induced tRNA pool would be calculated to have a low level of codon optimization—since this tRNA pool contains more rare codons which have frequencies that are very different from the background DNA.

We found further evidence for this effect in the heat-shock experiment. Although we did not observe a shift in the average level of optimization in the metatranscriptome, we did find a decrease in the average community-level of optimization for several upregulated genes. GroEL, groES, and ClpB, were all upregulated in response to a sudden temperature increase but averaged significantly lower levels of optimization at 60 °C than at 20 °C (*p* < 0.05). Although we cannot determine if a shift in the tRNA pool occurred, the abundance of stress-response genes with rare codons in the transcript pool in response to heat-shock is consistent with this hypothesis.

These results indicate that the relationship between codon usage and transcription may be context dependent. This is an important field of research, as a deeper understanding of the influence of codon usage in relation to gene expression under a variety of conditions might allow for better predictions of microbial behavior. For example, a gene with codon frequencies similar to sporulation genes might be transcribed and translated more efficiently under stress. Codon usage patterns of a genome could therefore serve as a predictive tool for assessing microbial responses under a broad range of environmental changes, including disturbance. Resistance and resilience are central to microbial community dynamics (Shade et al., 2012) and the ability to predict community response to disturbance from codon frequencies could be a valuable tool in metagenomic analyses.

## Conclusions

In this study, we expose several relationships between codon or nucleotide usage and the expression of genes in soil microbial communities. In response to the addition of glucose, we found that the total transcript pool had higher levels of codon optimization, energetically cheaper nucleotides at synonymous sites (*i.e*., lower GC and AT-skew), and encoded for energetically cheaper amino acids. However, these gene traits could not predict transcriptional expression either within a taxon or between taxa for a specific gene. We did find that when genes from a pathway were grouped, there was a positive relationship between codon optimization and average transcriptional expression between taxa. This represents a potential opportunity for linking specific process rates, such as nitrogen or carbon immobilization, to gene traits—similar to preexisting methods which use codon usage in ribosomal protein genes to predict growth (Weissman, Hou & Fuhrman, 2021). Future work should focus on examining protein production between soil communities with varied codon usage patterns in order to then link these features to biogeochemical processes. Together, our results demonstrate both the limitations and potential of codon usage, nucleotide frequency, and amino acid frequency in predicting microbial responses to environmental change.

Importantly, this work also highlights that the influence of gene traits on microbial activity in soils may be dependent on environmental context—adding deeper perspective to our understanding of functional potential. Codon usage has been referred to as a “code within the genetic code” (Liu, 2020), and other traits such as nucleotide selection and amino acid frequency could be seen through a similar lens. Determining how these embedded codes function in response to different environmental changes is critical towards understanding how microbes function in their environment. The presence of a gene is often only one component in determining the functional potential of an organism, which is ultimately shaped by the combination and interaction of several factors including (but not limited to) gene presence, regulatory networks, post-translational modifiers, environment, and gene traits. How specific stimuli interact with gene traits in a variety of contexts may therefore be an important consideration in bioengineering and synthetic community research, as well as in large-scale ecological analyses of the role of microbial communities in a changing world.

## Supplemental Information

10.7717/peerj.20641/supp-1 Supplemental Information 1 Supplemental tables and figures.

## Additional Information and Declarations

### DNA Deposition

The following information was supplied regarding the deposition of DNA sequences:

The raw sequence data are available at JGI GOLD: Gs0135756.

The raw sequence data are available at NCBI BioProject: PRJNA539715, PRJNA570412, PRJNA518697.

### Data Availability

The following information was supplied regarding data availability:

The code is available at Figshare: Chuckran, Peter (2025). Data and code for “The relationship between gene traits and transcription in soil microbial communities varies by environmental stimulus”. figshare. Dataset. https://doi.org/10.6084/m9.figshare.30446078.v1.

The MAG sequence data is available at FigShare: Chuckran, Peter (2025). sequence_data.zip. figshare. Dataset. https://doi.org/10.6084/m9.figshare.30100291.v1.

The raw sequence data are available at JGI GOLD: Gs0135756.
