# Supplementary material for: The relationship between gene traits and transcription in soil microbial communities varies by environmental stimulus
Source: PeerJ. 2026 Feb 5;14:e20641. doi: 10.7717/peerj.20641 (PMC12883164; doi:10.7717/peerj.20641)
Supplement: Supplemental Information 1 [file peerj-14-20641-s001.docx]

**Supplement Table 1**

Sample metadata for metagenomes and metatranscriptomes in temperature incubation experiment.

| **Temperature** | **Sample Type** | **Rep** | **IMG Genome ID** | **GOLD Analysis Project ID** | **NCBI Bioproject Accession** | **NCBI Biosample Accession** | **Gene Count** | **N**_50_ |
| --- | --- | --- | --- | --- | --- | --- | --- | --- |
| 20 | Metagenome | 1 | 3300034662 | Ga0314783 | PRJNA570395 | SAMN12813568 | 762561 | 1288729 |
| 20 | Metagenome | 2 | 3300034663 | Ga0314784 | PRJNA570396 | SAMN12814693 | 747033 | 880870 |
| 20 | Metagenome | 3 | 3300034479 | Ga0314785 | PRJNA570397 | SAMN12813706 | 108601 | 1913854 |
| 20 | Metagenome | 4 | 3300034661 | Ga0314782 | PRJNA570394 | SAMN12813049 | 896719 | 505047 |
| 60 | Metagenome | 1 | 3300034664 | Ga0314786 | PRJNA570398 | SAMN12812626 | 792202 | 1170933 |
| 60 | Metagenome | 2 | 3300031547 | Ga0310887 | PRJNA518696 | SAMN10864355 | 5152793 | 1009918 |
| 60 | Metagenome | 3 | 3300031943 | Ga0310885 | PRJNA539707 | SAMN11532793 | 4298207 | 1282903 |
| 60 | Metagenome | 4 | 3300032179 | Ga0310889 | PRJNA539708 | SAMN11532414 | 3727730 | 1194338 |
| 20 | Metatranscriptome | 1 | 3300031913 | Ga0310891 | PRJNA539710 | SAMN11532358 | 2019847 | 232158 |
| 20 | Metatranscriptome | 2 | 3300031538 | Ga0310888 | PRJNA518697 | SAMN10864145 | 5457649 | 38430 |
| 20 | Metatranscriptome | 3 | 3300034659 | Ga0314780 | PRJNA570392 | SAMN12814267 | 930641 | 243448 |
| 20 | Metatranscriptome | 4 | 3300031562 | Ga0310886 | PRJNA518695 | SAMN10864146 | 5429091 | 145253 |
| 60 | Metatranscriptome | 1 | 3300034660 | Ga0314781 | PRJNA570393 | SAMN12814181 | 583797 | 282854 |
| 60 | Metatranscriptome | 2 | 3300031944 | Ga0310884 | PRJNA539706 | SAMN11532342 | 5024730 | 174757 |
| 60 | Metatranscriptome | 3 | 3300034665 | Ga0314787 | PRJNA570399 | SAMN12813567 | 480705 | 271809 |
| 60 | Metatranscriptome | 4 | 3300032075 | Ga0310890 | PRJNA539709 | SAMN11532103 | 8416504 | 230787 |
|  |  |  |  |  |  |  |  |  |

**Supplemental Table 2**

The number of phosphate bonds used in synthesis, as described in (Akashi & Gojobori, 2002).

| **Amino acid** | **Symbol** | **Cost, ~P** |
| --- | --- | --- |
| Alanine | A | 11.7 |
| Arginine | R | 27.3 |
| Asparagine | N | 14.7 |
| Aspartic acid | D | 12.7 |
| Cysteine | C | 24.7 |
| Glutamic acid | E | 15.3 |
| Glutamine | Q | 16.3 |
| Glycine | G | 11.7 |
| Histidine | H | 38.3 |
| Isoleucine | I | 32.3 |
| Leucine | L | 27.3 |
| Lysine | K | 30.3 |
| Methionine | M | 34.3 |
| Phenylalanine | F | 52.0 |
| Proline | P | 20.3 |
| Serine | S | 11.7 |
| Threonine | T | 18.7 |
| Tryptophan | W | 74.3 |
| Tyrosine | Y | 50.0 |
| Valine | V | 23.3 |

Supplemental Figure 1:

Mean and standard error of nonsynonymous GC and AT skew in glucose-spike incubation.


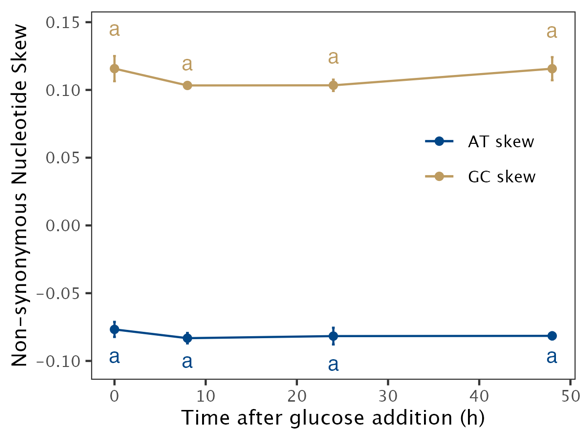


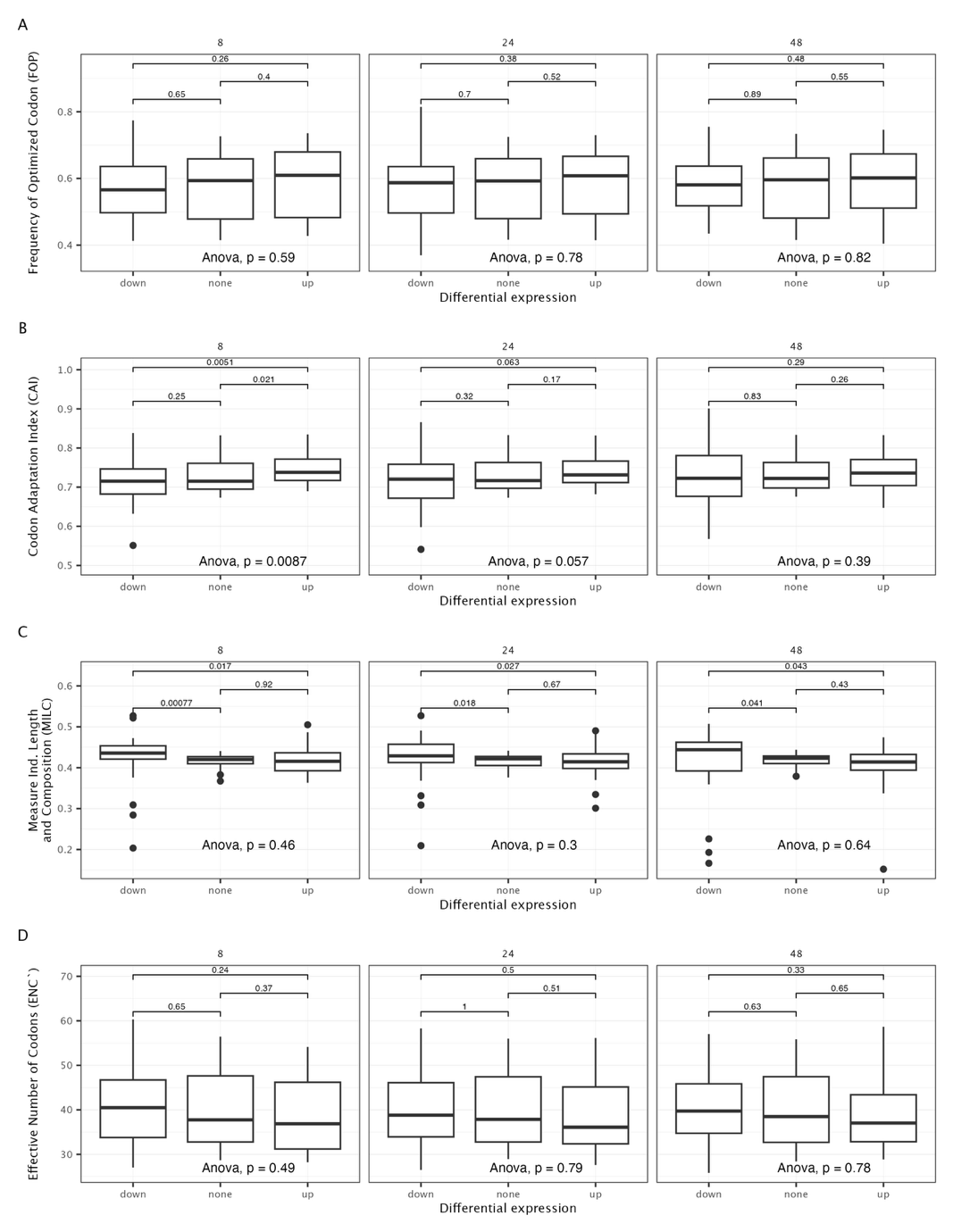
Supplemental Figure 2

Codon optimization of genes by differential expression, averaged by metagenome-assembled genome. Shown as **(A)** frequency of optimized codon, **(B)** codon adaptation index, **(C)** measure independent of length and composition, and **(D)** effective number of codons.


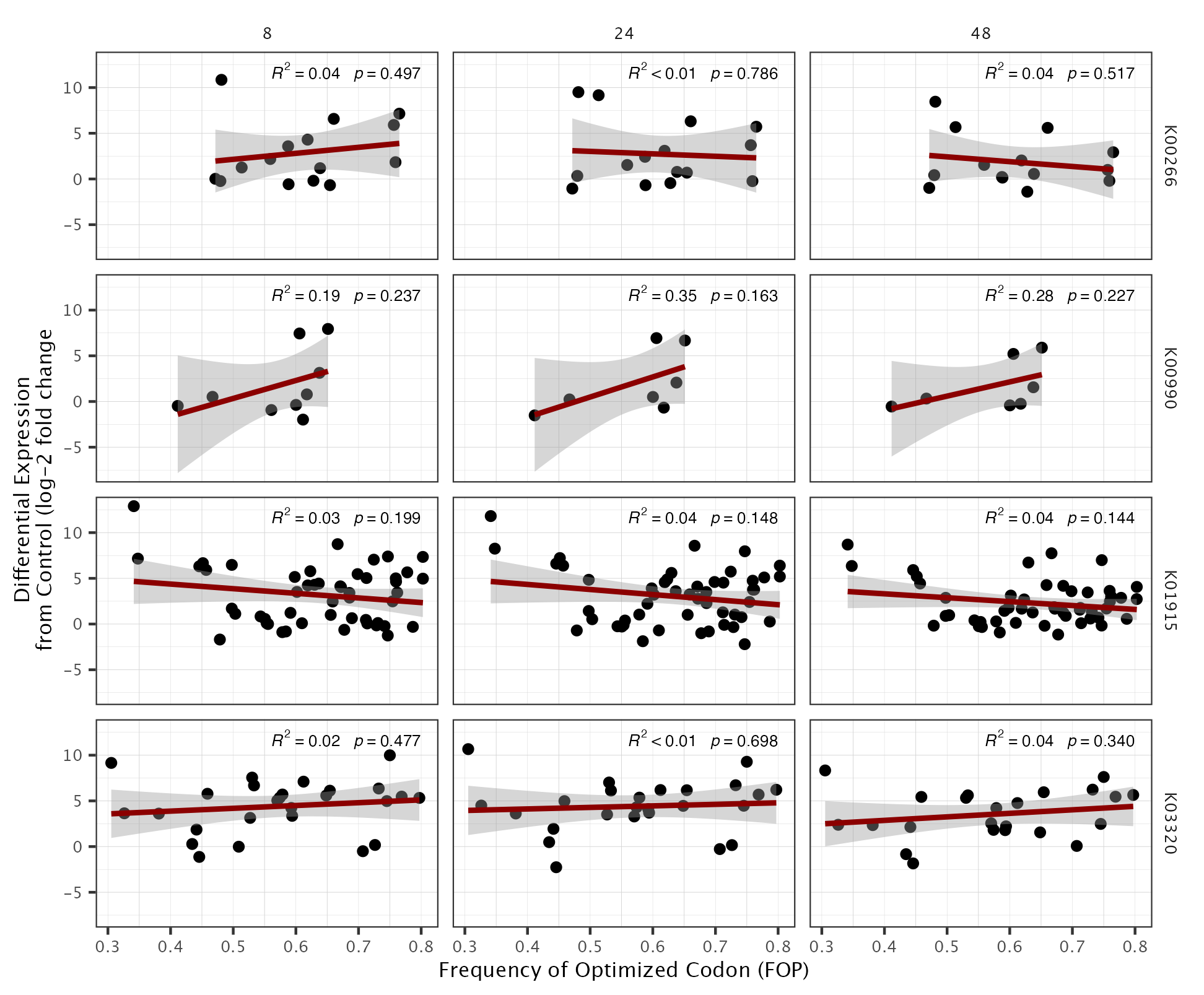
Supplemental Figure 3

Differential expression (log_2_-fold change) of select functional genes within MAGs in response to glucose amendments versus the frequency of optimized codon (FOP). Genes include *glnA* (K01915), *gltD* (K00266), amt (K03320), and *glnD* (K00990).


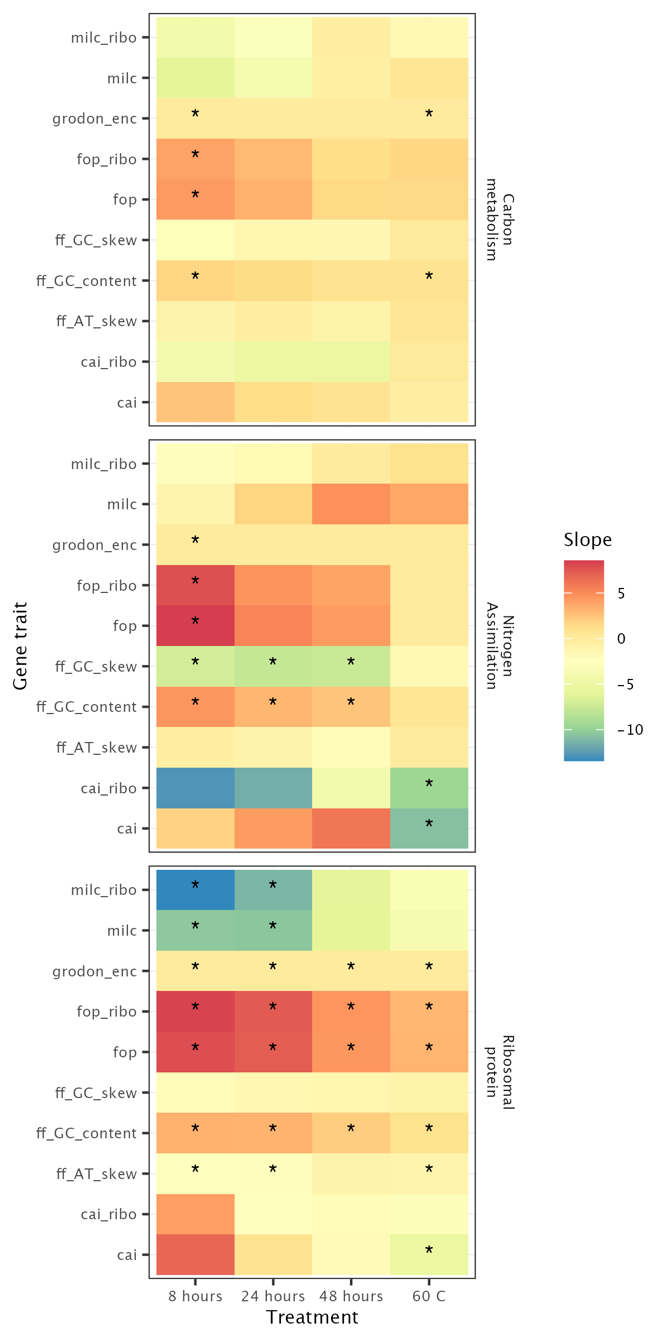
Supplemental Figure 4

The relationship between gene traits and expression (log_2_-fold change) for select functions in metagenome assembled genomes. Color represents the slope between gene traits and log_2_-fold change versus control. Asterisk indicates regressions with a p-value of < 0.05. Traits with the suffix “_ribo” indicate gene traits where the background codon usage used in calculations was based on ribosomal protein genes. The prefix “ff_” indicates that the trait refers to fourfold degenerative sites (i.e. the third nucleotide on codons where all nucleotide substitutions code for the same amino acid).
